# Association of Genetic Polymorphism rs 77630697(Gly64Asp) of Multidrug and Toxin Extrusion -1 with glycemic response to metformin in patients with Type 2 Diabetes Mellitus

**DOI:** 10.12669/pjms.40.6.8170

**Published:** 2024-07

**Authors:** Zunera Hakim, Najam ul Hasan, Asma Khan, Akbar Waheed

**Affiliations:** 1 Zunera Hakim, MPhil. Najam ul Hasan, FCPS. Department of Pharmacology, Rawalpindi Medical University, Rawalpindi, Pakistan; 2 Najam ul Hasan, FCPS. Department of Ophthalmology, PAF Hospital, Shorkot, Pakistan; 3 Asma Khan, MPhil. Department of Pharmacology, Rawalpindi Medical University, Rawalpindi, Pakistan; 4 Akbar Waheed, PhD. Department of Pharmacology Islamic International Medical College, Rawalpindi, Pakistan

**Keywords:** Metformin, Polymorphism, Diabetes Mellitus

## Abstract

**Objective:**

To determine the relationship between Gly64Asp (rs77630697) polymorphism of multidrug and toxin extrusion-1 (MATE-1) and therapeutic response of metformin in Type-2 diabetic patients.

**Methods:**

A longitudinal study was conducted at Riphah International Hospital, Islamabad from June 2020 to December 2021. Type-2 diabetic patients (n=200) on metformin monotherapy fulfilling the inclusion criteria were enrolled and followed up till three months. Based on change in HbA1c, they were divided into responders and non-responders. DNA was extracted and genotyping was done by TETRA ARMS PCR. Data was entered and association was analyzed by SPSS 22.

**Results:**

Out of 200 participants, 104 were categorized as responders and 96 as non-responders. The genotype and allelic distribution of rs77630697 was significantly different between responders and non-responders. The variant genotype (GG) was most prevalent among the study population and among responders. After follow up of three months, difference in glycemic response was found to be statistically significant (*p* < 0.05) among three genotypes (GG, GA and AA). The decline in HbA1c was highest in GG genotype with almost two-fold reduction in comparison with GA and AA. Carriers of allele A were significantly associated with impaired response to metformin.

**Conclusion:**

The variable therapeutic response to metformin in the responders and non-responders may be contributed to rs77630697 isoform variation of MATE-1.

## INTRODUCTION

Type-2 Diabetes Mellitus (T2DM) has become a global health challenge as its prevalence continues to increase. International Diabetic Federation (IDF) has reported that T2DM currently affects 462 million people worldwide and its incidence is expected to increase to 642 million by 2040.^1^ With 33 million cases, Pakistan presently holds third highest T2DM prevalence in the world.^2^

Diabetes can be managed by early diagnosis, life style modifications and pharmacotherapy with different anti-diabetic drugs. Many international organizations have recommended metformin as the first line treatment of T2DM due to its effectiveness, low cost and safety profile.^3^

However, about 35% patients on metformin monotherapy fail to respond optimally.^4^ This difference in metformin efficacy can be due to both genetic and non-genetic factors. While phenotypic characteristics like age, gender, weight contribute only partially to this inter-individual difference, 90% of the variability can be attributed to genetic variants.^5^ These genetic variants represented by SNPs affect the therapeutic efficacy of metformin by regulating the pharmacokinetics and pharmacodynamics of metformin.

Many pharmacogenetic studies have explored the influence of several genes (*SLC22A1, SLC22A2, SLC22A3, SLC29A4, SLC47A1 & SLC47A2*) that can regulate the response of metformin.^6^ Since metformin is a positively charged molecule, these genes mostly code for different membrane transporters (organic cation transporters, plasma membrane monoamine transporter and multidrug and toxin extrusion transporters )^7^ required for its distribution and elimination.

MATE family also called the SLC 47 family are efflux transporters characterized into MATE-1 and MATE2-K encoded by genes *SLC47A1* and *SLC47A2*, present on 17^th^ chromosome (17p11.2). MATE-1 is highly expressed on the canalicular membrane of hepatocytes and proximal renal tubules. MATE2-K is specifically present on brush border of renal epithelium. The renal elimination of metformin is carried out by both MATE-1 and MATE-2K.^8^

Genes encoding MATE-1 are polymorphic and can lead to altered transporter function which could subsequently influence the glucose lowering effect of metformin.^9^,^10^ Thus, polymorphic variants of *SLC47A1* can play an important role in interethnic and inter-individual variation in glycemic response of metformin. Unfortunately, limited global studies have explored only a few MATE-1 variants and provided inconsistent results. In Pakistan, studies demonstrating the impact of *SLC47A1* polymorphism on metformin effectiveness in diabetic patients are exigous. However, only one prior study conducted in Pakistani population has highlighted the effect of this transporter gene on responder and non-responder ratio in our region.^11^ Thus, the main objective of our study was to explore the genotypic and allelic frequencies of SNP rs77630697 on *SLC47A1* and to investigate the relationship between this genetic variant and metformin efficacy among T2DM patients on metformin monotherapy.

## METHODS

A longitudinal analytical study was carried out in Riphah International Hospital, Medicine Department in collaboration with the Department of Pharmacology and Therapeutics, Islamic International Medical College, Rawalpindi from June 2020 to December 2021.

### Ethical Approval:

The research was conducted after approval from the Ethical Review Board of the institute (Appl.# Riphah/IRC/18/0280) in accordance with the current Good Clinical Practices and the Declaration of Helsinki.

### Inclusion & Exclusion Criteria:

Type-2 unrelated patients of either gender, aged between 35 and 70 years, treated with metformin for three consecutive months in the diabetic clinic were enrolled in the study after written informed consent. Patients were clinically diagnosed according to criteria of American Diabetic Association.^12^ Exclusion criteria was type-I diabetes, pregnant and lactating women, hepatic, renal (creatinine clearance >1.5mg/dL in male and >1.4mg/dL in female) and cardiac abnormalities and individuals on concomitant medications that act as substrates or inhibitors of MATE transporters(cimetidine, famotidine, pyrimethamine etc.).^13^ Sample size of 216 patients was calculated employing the WHO sample size calculator. However, due to later stage drop out and non-compliance with treatment, it was reduced to 200 participants.

At the time of induction, all relevant baseline characteristics such as gender, age, weight, height, BMI and creatinine levels were documented. Venous blood was withdrawn from each participant under sterile conditions for HbA1c evaluation and genotyping. HbA1c levels were taken twice in the project once at the initiation of metformin and then after completion of three months of therapy. Metformin monotherapy was started at 500mg twice daily and titrated up to a maximum of 2000mg/day over a period of 12 weeks with follow up visits at 4, 8 and 12 weeks. Dose titration was done on the basis of glycemic control at each visit. Based upon the reduction in HbA1c from baseline, the participants were divided into metformin responders (decrease in HbA1c levels by more than 0.8% from the baseline) and non-responders (decrease in HbA1c levels by less than 0.8%).^14^ HbA1c was quantified by Bio-Rad D-10 Hb testing system which uses the HPLC ion exchange for determination of glycated hemoglobin in the sample.

SNP rs77630697 of *SLC47A1* encoding MATE was selected based on its location on exon-2 affecting the structure and transport activity of MATE-1 thus influencing the response of metformin. DNA was extracted from blood using chelex solution and then stored for SNP rs 77603697 analysis. TETRA ARMS PCR was used with four primers with the sequence.

RS97 FI-G: TCAGCTTCATAAGCTCCGTGTTCTGGGG

RS97 RI-A: ATCCAGCTCCAGCTTGCCCAGGTTGT

RS97 FO: TCCCCAGCCCCAGAAATTGTACATTTGC

RA97 RO: AACCCACTTCAGACTTCTGCACTCCAGCA

The reaction mixture was then processed with thermal stages including initial denaturation at 98ºC for three minutes followed by 35 cycles of 30 seconds each of the temperatures 98ºC, 63ºC, and 72ºC. Final extension was carried out at 72ºC for seven minutes. Amplified products were visualized via 2% agarose gel electrophoresis.

### Statistical Analysis:

The SPSS version 22 was used to analyze statistical data. Mean ± S.D was used to depict quantitative data. The independent and paired samples’ *t-test* was employed to compare differences between continuous variables. The chi square test was applied to examine the difference in the genotypic and allelic frequencies of rs77630697 between metformin responders and non-responders. Intergroup differences across various genotypes were calculated using one-way analysis of variance (ANOVA) with the multiple comparison post hoc Tukey test. The association of metformin responsiveness with rs77630697 variations was investigated using binary logistic regression. Statistical significance was defined as a *p*-value < 0.05.

## RESULTS

**Fig.1:**
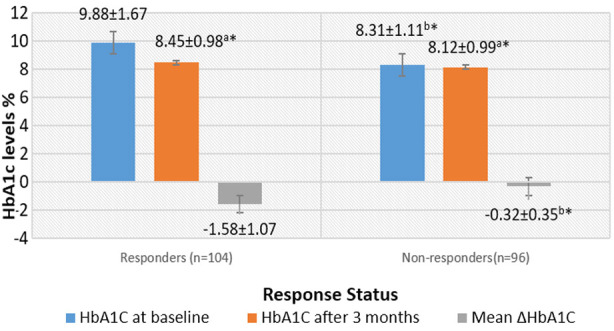
Changes in mean HbA1c values at baseline and after metformin therapy in responders and non-responders (n=200). *p>0.05 is significant a* when baseline HbA1c compared with HbA1c at three months (paired sample *t* test) b* when HbA1c levels compared between responders and non-responders (independent sample *t* test).

The general clinical characteristics of 200 recruited individuals are given in Table-I. On the basis of response to metformin, 104 (52%) patients were classified as responders whereas the remaining 96(48%) patients fail to respond optimally. Responders and non-responders show significant difference between HbA1c levels at baseline and after three months. The decrease in mean HbA1c levels was much higher (statistically significant) in responders than non-responders. (Fig.1).

**Table-I:** Baseline parameter of study participants.

Parameters	Total (n=200)	Metformin responders (n=104)	Metformin non-responders (n=960	p-value
Age (years)	46.25 ±9.02	48.42±8.91	44.06±7.64	0.00*^a^
***Gender***				0.60^b^
Female	115	58(51%)	57(49%)	
Male	85	46(54%)	39(46%)	
Weight (kg)	66.54±10.22	68.45±9.84	64.45±10.17	0.00*^a^
Height (m^2^)	2.17±0.36	2.25±0.25	2.18±0.24	0.02*^a^
BMI (kg/m^2^)	29.76±5.77	30.55±4.07	29.85±4.75	0.26^a^
Creatinine level (mg/dL)	0.87 ±0.20	0.90±0.19	0.86±0.18	0.19^a^

Significant

* p value: < 0.05

a Independent sample t-test,

b Pearson Chi square test.

### Frequencies of rs 77630697 polymorphism in study participants:

All the patients were genotyped for SNP rs 77630697 which was found to be in agreement with Hardy Weinberg equilibrium. The distribution of allele and genotype frequencies between metformin responders and non-responders was statistically significant as shown in Table-II.

**Table-II:** Gene and allelic frequencies of SLC47A1 rs 77630697 polymorphism in metformin responders and non-responders.

Genotype		Response status		Total (n=200)	^↨^p value

		Responder (n=104)	Non-responder (n=96)		
SNP	GG	64(61.5%)	42(43.8%)	106 (53%)	0.039*
	GA	32(30.8%)	45(46.9%)	77(38.5%)	
	AA	8 (7.7%)	9(9.4%)	17 (8.5%)	
Allele	G	160 (76.9%)	129(67.2%)	289(72.2%)	0.030*
	A	48 (23.1%)	63 (32.8%)	111(27.8%)	

Significant

* p value: < 0.05

↨ Pearson Chi square test.

The baseline HbA1c was not significantly different among the three genotypes of *SLC47A1*.After follow up of three months, all the genotypes showed significant lower level of HbA1c than those in baseline (p<0.05). Follow up HbA1c levels and the mean change in HbA1c were found to be statistically different across three variants of *SLC47A*1 gene, (Table-III). Association studies showed significant association of genotype GA with metformin therapy. Patients with one mutant allele “A” have higher odds of not responding to metformin when compared with GG. However, genotype AA was found to have no association with efficacy of metformin in the study population (Table-IV).

**Table-III:** Comparison of clinical response to metformin across SLC47A1 genotypes.

	GG	GA	AA	p value
HbA1c baseline	9.24±1.64	9.07±1.46	9.43±1.49	0.62
HbA1c after 3 months	^a,b^8.08±1.03	^a^8.28±1.08	^a^8.73±0.987	0.04
Mean ∆HbA1c	^C^-1.16±1.11	-0.785±0.799	-0.700±01.00	0.02

^a.^ p< 0.05 with HbA1c baseline, ^b.^ p < 0.05 compared with AA, ^c.^ p <0.05 with GA group.

**Table-IV:** rs 77630967 Polymorphism Association in Metformin Responders and Non Responders.

Genotype	Metformin responder	Metformin Non-responder	β	OR	95% CI	p value
GG	64	42		1 (Reference)		
GA	32	45	-0.762	0.467	0.257-0.848	0.012*
AA	8	9	-0.539	0.583	0.208-1.632	0.305

Significant

* p <0.05.

## DISCUSSION

Achievement of glycemic control is one of the main goals of successful pharmacological management of T2DM. However, genetic determinants modulating glycemic response to metformin makes it difficult to achieve desired targets. In our study, we reported the role of MATE-1 variants on metformin efficacy which is one of the pioneering studies done among Pakistanis. The effectiveness of any antidiabetic drug is based on its magnitude of reduction in HbA1c. Clinical studies have demonstrated that oral anti diabetic drugs produce an estimated decrease of 0.5-1.5% in HbA1c levels.^15^ Similar to this, in our project, HbA1c levels decreased in average by -0.97±1.01% after three months of metformin monotherapy. With a cut off value of ≥0.8% reduction in HbA1c levels (from baseline) as a response to metformin therapy^14^,104 (58 females and 46 males) subjects responded optimally while 96 (57 females and 39 males) did not. Response status of our participants is similar to an Iranian study with 51% responders and 49 % as non-responders.^16^ Baseline HbA1c levels in the non-responder group were significantly lower than those in the responder group (8.31±1.11% VS 9.88±1.67). Similarly, the higher baseline HbA1c values observed in the responder group significantly decreased by 15 % after three months of metformin therapy. This was also reflected in the statistically greater mean change in HbA1c (post vs pre-treatment) in responders than that in non-responders (-1.58± 1.07 VS -0.32±0.35). This supports the documented positive association between higher baseline A1c levels and greater reduction in HbA1c with metformin.^17^,^18^

Lack of sufficient evidence showing a possible association between MATE-1 polymorphism and metformin treatment outcomes sparked interest to pursue this research. Kajiwara and his fellows, first identified five non-synonymous SNPs (V10L, G64D, A310V, D328A and N474S) in MATE-1 that exhibited a significant decrease in transport activity.^19^ We decided to investigate the effect of one of these unexplored variants (rs 77630697, MATE-1 G64D) in the *SLC47A1* gene on metformin therapy in T2DM patients.

In the current study, we found a significant association between the rs77630697 variant of the *SLC47A1* gene and its effectiveness in reducing HbA1c concentrations in the patients. According to the 1000 Genome Project, the minor allele frequency (MAF) in the studied population was 0.27 which was much higher than the MAF in other populations; 0.0065 in East Asians and Asians whereas 0.0000 in Americans, European and Africans respectively (https://www.ncbi.nlm.nih.gov/snp/rs77630697). In their investigation, Sadaf Moeez and her colleagues also noted that the examined variant in the Pakistani population had an equivalently higher MAF (0.25).^11^

According to our findings, patients had a greater frequency of allele G of rs 77630697 variant with GG genotype being predominant followed by GA and AA. It was further demonstrated that carriers of two copies of allele G(GG) of this SNP were more likely to respond satisfactorily to metformin. Carriers of one minor allele ‘A’ (GA) were less common among metformin responders than among those who failed to respond (30.8% VS 46.9%). The AA genotype was higher in (9.4% VS 7.7%) metformin non-responders. This backs up the findings of a study done on 800 Pakistani people, where 62% of responders carried the GG allele, while GA and AA (51%) were the most common genotypes among non-responders.^11^

Patients carrying homozygous GG variant of rs 77630697 had noticeably superior glucose lowering effect after three months of metformin monotherapy. Individuals with the genotype GG showed a statistically larger drop in HbA1c levels (12 % reduction from baseline) than did those with the GA (8% reduction from baseline) and AA genotypes (7% reduction from baseline). This was also reflected in the average change in HbA1c levels which was more pronounced in GG genotype. These results are consistent with the published study suggesting that metformin is therapeutically more effective in T2DM patients with GG genotype of *SLC47A1*.^11^,^20^ The association of genotypic variants with response of metformin revealed that Pakistani carriers of minor allele (A) were more likely to be non-responders and exhibit unsatisfactory glycemic control. Prior Pakistani based population study, also deduced that patients carrying copies of allele ‘G’ (GG) had two-fold increased chance to achieve therapeutic response to metformin.^11^,^20^ Thus, variants in transporter genes may determine the clinical efficacy of metformin.

This study has recognized the predictive role of this new variant of MATE-1 on metformin response. This also adds valuable information to the only prior human study conducted on this SNP with respect to therapeutic efficacy of metformin. Limited available literature has mainly studied the effect of rs 77630697 on cell lines. Identification of novel genetic determinants of metformin glycemic effect will help in stratifying patients by levels of response and tailoring metformin therapy to those most likely to benefit from it.

### Limitations of the study:

A smaller sample size and lack of effect of MATE-1 variant on the pharmacokinetic parameter can be considered a limitation.

## CONCLUSION

In this study we have demonstrated that rs 77630697 of *SLC47A1* may affect the clinical outcome of metformin in T2DM patients. Our data suggests that patients with the GG genotype are more likely to get maximum glucose lowering response from metformin therapy.

### Recommendations

1. Studies with larger sample size in different ethnic groups.
2. Joint investigation with multiple transporters involved in pharmacokinetics of metformin.

### Authors Contribution:

**ZH:** Conceived, designed, drafted manuscript and did statistical analysis, is responsible for integrity of research. **NH:** Analyzed data and edited manuscript. **AK:** Analyzed, interpreted data and did critical revision. **AW:** Reviewed and gave final approval of manuscript.
